# The Diagnostic Accuracy of an Electrocardiogram in Pulmonary Hypertension and the Role of “R V1, V2 + S I, aVL − S V1”

**DOI:** 10.3390/jcm13247613

**Published:** 2024-12-13

**Authors:** Lukas Ley, Christoph B. Wiedenroth, Stefan Guth, Christian Gold, Athiththan Yogeswaran, Hossein Ardeschir Ghofrani, Dirk Bandorski

**Affiliations:** 1 Campus Kerckhoff, Justus Liebig University Giessen, 61231 Bad Nauheim, Germany; lukas.m.ley@med.uni-giessen.de; 2 Kerckhoff Heart and Thorax Center, Department of Thoracic Surgery, 61231 Bad Nauheim, Germany; c.wiedenroth@kerckhoff-klinik.de (C.B.W.); s.guth@kerckhoff-klinik.de (S.G.); 3 Department of Cardiology and Vascular Medicine, University Hospital Frankfurt, 60596 Frankfurt am Main, Germany; christian_gold@web.de; 4 Department of Internal Medicine, Justus-Liebig-University Giessen, Universities of Giessen and Marburg Lung Center (UGMLC), 35392 Giessen, Germany; athiththan.yogeswaran@innere.med.uni-giessen.de (A.Y.); ardeschir.ghofrani@innere.med.uni-giessen.de (H.A.G.); 5 Kerckhoff Heart and Thorax Center, Department of Pneumology, 61231 Bad Nauheim, Germany; 6 Department of Medicine, Imperial College London, London SW7 2AZ, UK; 7 Faculty of Medicine, Semmelweis University Campus Hamburg, 20099 Hamburg, Germany

**Keywords:** pulmonary hypertension, electrocardiogram, diagnostic accuracy, R V1, V2 + S I, aVL − S V1

## Abstract

**Background**: Pulmonary hypertension (PH) can cause characteristic electrocardiographic (ECG) changes due to right ventricular hypertrophy and/or strain. The aims of the present study were to explore the diagnostic accuracy of ECG parameters for the diagnosis of PH, applying the recently adjusted mean pulmonary artery pressure (mPAP) threshold of >20 mmHg, and to determine the role of “R V1, V2 + S I, aVL − S V1”. **Methods**: Between July 2012 and November 2023, 100 patients without PH, with pulmonary arterial hypertension, or with chronic thromboembolic pulmonary hypertension were retrospectively enrolled. **Results**: The sensitivity and specificity of the ECG parameters for the diagnosis of PH varied from 3 to 98% and from 3 to 100% (means: 39% and 87%). After optimising the parameters’ cut-offs, the mean sensitivity (39% to 66%) increased significantly but the mean specificity (87% to 74%) slightly decreased. “R V1, V2 + S I, aVL − S V1” was able to predict an mPAP >20 mmHg (OR: 34.33; *p* < 0.001) and a pulmonary vascular resistance >5 WU (OR: 17.14, *p* < 0.001) but could not predict all-cause mortality. **Conclusions**: Even with improved cut-offs, ECG parameters alone are not able to reliably diagnose or exclude PH because of their low sensitivity. However, they still might be helpful to reveal a suspicion of PH, especially in early diagnostic stages, e.g., in primary care with general practitioners or non-specialised cardiologists and pulmonologists. “R V1, V2 + S I, aVL − S V1” was able to predict the diagnosis of (severe) PH but could not predict all-cause mortality. Nevertheless, it can still be useful in risk stratification.

## 1. Introduction

Pulmonary hypertension (PH) is a cardiopulmonary disease which, in most cases, causes distressing symptoms, a reduced quality of life, and a relevant mortality rate [1,2,3,4]. PH is classified into five groups based on the underlying pathomechanism [1]. Pulmonary arterial hypertension (PAH, group 1) and chronic thromboembolic pulmonary hypertension (CTEPH, group 4) are less common groups but can be treated by specific drugs, surgery, or interventions. The fundamental tools used to diagnose PH are the echocardiogram as a screening tool and right heart catheterisation (RHC) as the gold standard for the haemodynamic confirmation of PH (mean pulmonary artery pressure (mPAP) > 20 mmHg) [1,2]. However, PH can also cause characteristic electrocardiographic changes due to right ventricular hypertrophy (RVH) and/or strain [5]. These electrocardiographic changes have proven helpful in establishing or ruling out the diagnosis and predicting mortality [5,6,7,8]. Moreover, some distinct electrocardiographic parameters, e.g., “R V1, V2 + S I, aVL − S V1”, proved helpful in establishing a PH diagnosis, correlated with cardiopulmonary haemodynamics and RVH, and were able to hint a worse prognosis and a therapeutic improvement in cardiopulmonary haemodynamics [8,9,10,11]. However, the electrocardiogram (ECG) does not play a major role in the current European Society of Cardiology (ESC)/European Respiratory Society (ERS) guidelines, and previous studies described a low sensitivity (range: 3–93%) and low negative predictive value (NPV, range: 9–100%) but a relatively good specificity (range: 50–100%) and positive predictive value (PPV, range: 0–100%) for ECG parameters for the diagnosis of PH or the detection of indirect PH signs, such as right atrial enlargement or RVH [1,6,9,12,13,14,15,16,17,18,19]. Since the ECG is a rapid, simple, non-invasive, and ubiquitously available test, the consistent use of it, if helpful, would be desirable in the diagnostic process of PH [5]. Because ECGs are performed in many clinical situations and usually at the beginning of the PH diagnostic algorithm, they could potentially help to recognise PH earlier. This is desirable, as PH is often diagnosed with a significant delay (with over one year from symptom onset to PH diagnosis often due to the consultation of several physicians being necessary), which is associated with increased right heart strain and potentially worse prognosis [1,2,20,21,22,23,24]. However, because the transferability of the results of previous diagnostic accuracy studies is limited due to the application of the old mPAP threshold (≥25 mmHg), the aim of the present study was to extensively explore the diagnostic accuracy of ECG parameters for the diagnosis of PH by applying the new mPAP threshold of >20 mmHg, which is now no longer arbitrarily set but based on scientific data [25,26]. Moreover, the present study aimed to explore potentially more appropriate and effective cut-offs for currently used electrocardiographic parameters and to define the role of “R V1, V2 + S I, aVL − S V1” in diagnosing PH and predicting mortality.

## 2. Materials and Methods

### 2.1. Study Design

The present study was conducted as a bicentric, retrospective study in two German high-volume referral centres for PAH and CTEPH. Three groups of patients were included between July 2012 and November 2023. The control group (CG) consisted of consecutive patients with suspected CTEPH but in whom PH was ruled out by RHC (mPAP ≤ 20 mmHg). The PAH and CTEPH groups consisted of consecutive patients with newly diagnosed PAH or CTEPH (mPAP > 20 mmHg). The patients were retrospectively reviewed regarding the inclusion criteria (PH exclusion or PAH/CTEPH diagnosis according to the 2022 ESC/ERS guidelines, sufficient RHC and electrocardiographic data available, and available written informed consent to participate in the study) and included if they met the criteria. All patients with PAH were also enrolled in the “Giessen Pulmonary Hypertension Registry and Biobank” (NCT04145024). All patients with CTEPH were also enrolled in the New International CTEPH Database (NCT02656238), and some were enrolled in the International BPA Registry (NCT03245268) in ongoing studies within the Collaborative Research Center (CRC1213) or previous publications [27,28].

### 2.2. Electrocardiogram

Twelve-lead ECGs (MAC 1200 ST, GE Medical Systems, Chicago, IL, USA and Cardiovit AT-10, SCHILLER Medizintechnik, Feldkirchen, Germany) were recorded (paper speed: 50 mm/s; sensitivity: 10 mm/mV) at the time of ruling out or the diagnosis of PH with patients in the supine position. ECGs were analysed with standard nomenclature and definitions by two physicians blinded to the patients’ clinical characteristics and outcomes. However, the ECGs were primarily analysed by only one author, and a second author, an experienced cardiologist, was only consulted in case of uncertainties. The selected electrocardiographic parameters (Table S3) are commonly used ECG parameters in clinical routine or are typical and validated ECG parameters of right heart strain and RVH, which have already been used in previous studies [6,8,11,12,29,30,31,32,33,34,35,36,37,38,39,40,41,42,43,44,45,46,47]. The electrocardiographic main parameters (EMPs, Table S2) were selected based on the results of a previous study. In this previous study, the EMPs occurred with a comparatively high prevalence (15–57%), so it was hypothesised that they might reveal a relatively high sensitivity. Moreover, most of them were relatively easy to detect and could therefore be conveniently applicable in clinical practice. The parameter “R V1, V2 + S I, aVL − S V1” and its cut-off (>0.6) were chosen for a more detailed analysis as they seemed to be able to indicate clinically and haemodynamically more severe disease as well as an increased risk of death in a previous study [8]. Table S7 provides some explanations on the calculation and use of some of the more complex electrocardiographic parameters applied.

### 2.3. Right Heart Catheterization

RHC was performed according to current guidelines and the standard operating procedure to confirm or exclude PH. A 7 French Swan–Ganz catheter (e.g., Thermodilution Catheter TD1704NX, Bioptimal, Singapore) was advanced into a pulmonary artery by puncturing the internal jugular vein using Seldinger’s technique. Pressure was measured in the right atrium (right atrial pressure: RAP) and the pulmonary artery (pulmonary artery pressure: PAP). mPAP was calculated from systolic (sPAP) and diastolic PAP (dPAP). Cardiac output (CO) and cardiac index (CI) were measured and calculated using the thermodilution method. Pulmonary vascular resistance (PVR) was calculated as follows: (mPAP—pulmonary capillary wedge pressure) × 80/CO. Severe PH was defined as PVR > 5 Wood Units (WU) or PVR > 400 dyn∗sec∗cm^−5^, respectively.

### 2.4. Statistics

A statistical analysis was performed with SPSS Statistics (Version 29, IBM, Armonk, NY, USA) and Jamovi (Version 2.3, Sydney, Australia). Nominal variables are presented as numbers and percentages. Since the Shapiro–Wilk test revealed that almost every variable was non-normally distributed, continuous variables are presented as median and interquartile range. To compare variables between different groups, Fisher’s exact test for nominal variables and Mann–Whitney U test for continuous variables were performed. Due to multiple testing, Bonferroni correction had to be applied for the EMPs, and a *p*-value < 0.004 was considered to be statistically significant. Sensitivity, specificity, PPV, and NPV of electrocardiographic parameters for the diagnosis of PH were calculated according to the following assumptions: the presence of electrocardiographic parameters in patients with PH was considered a true positive event, and the absence of electrocardiographic parameters in patients without PH was considered a true negative event. The absence of electrocardiographic parameters in patients with PH was considered a false negative event, and the presence of electrocardiographic parameters in patients without PH was considered a false positive event. The optimum cut-offs, their sensitivity and specificity, and the area under the curve (AUC) were determined by a receiver operating characteristic (ROC) analysis and the Youden index. Univariate and multivariate logistic regression analyses were used to identify predictors of PH among the EMPs. Univariate logistic regression analysis was also used to identify “R V1, V2 + S I, aVL − S V1” as a possible predictor of (severe) PH and consecutive mortality. A Kaplan–Meier curve was created, and a log-rank test was performed to detect potential differences in survival between the groups over (>0.6 mV) and under (≤0.6 mV) a defined cut-off for “R V1, V2 + S I, aVL − S V1”.

## 3. Results

A total of 300 patients, subdivided into three groups (CG, PAH group, and CTEPH group) containing 100 patients each, were included in the study. The CG, PAH, and CTEPH groups consisted of 55%, 57%, and 52% women, and the median ages were 60.8, 65.7, and 63.2 years, respectively. Haemodynamic, clinical, and functional data can be found in Table 1 and Table S1.

The prevalence of the EMPs in the CG was low (0–15%). The EMPs were significantly more frequent in the PAH (13–64%) and CTEPH (14–57%) groups. In addition, the EMPs occurred more frequently in severe PH (17–69%) than in non-severe PH (0–35%). Further ECG data can be found in Tables S2 and S3.

The sensitivity, specificity, PPV, and NPV of electrocardiographic parameters for the diagnosis of PH varied in the ranges of 3–98%, 3–100%, 60–100%, and 22–54% with mean values of 39%, 87%, 90%, and 42%. The sensitivity, specificity, PPV, and NPV of the EMPs for the diagnosis of PH varied in the ranges of 14–61%, 85–100%, 86–100%, and 36–54% with mean values of 40%, 96%, 95%, and 45% (Table 2).

In severe PH, the mean sensitivity (42% vs. 21%) and mean PPV (87% vs. 53%) were higher, and the mean NPV was lower (49% vs. 72%) than in non-severe PH (Table S4).

The currently applied cut-offs of all analysed ECG parameters were found to be not optimal for PH diagnosis and were adjusted. After optimisation, the sensitivity (range: 3–98% to 29–81%; mean: 39% to 66%) and Youden index (range: −0.18–0.53 to −0.22–0.68; mean: 0.26 to 0.40) increased significantly, but specificity (range: 3–100% to 37–92%; mean: 87% to 74%) slightly decreased (Table S5).

In the univariate logistic regression analysis, 12/13 of the EMPs were able to predict an mPAP > 20 mmHg (Table 3). In the multivariate logistic regression analysis, only the parameters “QRS axis associated with right heart strain” (OR (odds ratio): 6.10; 95% confidence interval (95% CI): 2.16–17.28; *p* = 0.006) and “Time to R peak in V1 (QRS < 120 ms) > 35 ms” (OR: 3.69; 95% CI: 1.45–9.38; *p* < 0.001) remained independent predictors of an mPAP > 20 mmHg.

A total of 51% of patients with PH exceeded a defined cut-off (0.6 mV) of the parameter “R V1, V2 + S I, aVL − S V1”. The percentage of men represented in this group was higher (55% vs. 35%; *p* = 0.005) and the median age was lower (62.4 vs. 68.9 years; *p* = 0.015). These patients were more likely to have severe PH (93% vs. 67%; *p* < 0.001) and presented with a higher median mPAP (47 vs. 40 mmHg; *p* < 0.001) and PVR (684 vs. 510 dyn∗sec∗cm^−5^; *p* < 0.001). Moreover, these patients with PH had higher brain natriuretic peptide (BNP, 391 vs. 189 pg/mL; *p* = 0.003) and N-terminal (NT)-pro-BNP levels (1108 vs. 210 pg/mL; *p* < 0.001). In addition, the patients with PAH who exceeded this cut-off were more frequently categorised as high risk (three-stratum risk assessment model) at the time of diagnosis (24% vs. 6%; *p* = 0.023). However, the distributions of the New York Heart Association (NYHA) class and 6 min walk distance (6MWD) were not significantly different. A total of 12/13 of the EMPs were statistically significantly more frequent in the exceedance group (19–100% vs. 0–46%; *p* < 0.001–*p* = 0.013). Further data can be found in Table S6.

However, the currently applied cut-off (>0.6 mV) did not seem to be optimal for the diagnosis of PH (sensitivity: 52%; specificity: 97%). A new cut-off (>0.12 mV) was able to diagnose PH with sensitivity and specificity values of 75% and 80% (AUC: 0.84; accuracy: 0.80). Therefore, the Youden index increased (0.49 to 0.55).

**Figure 1:**
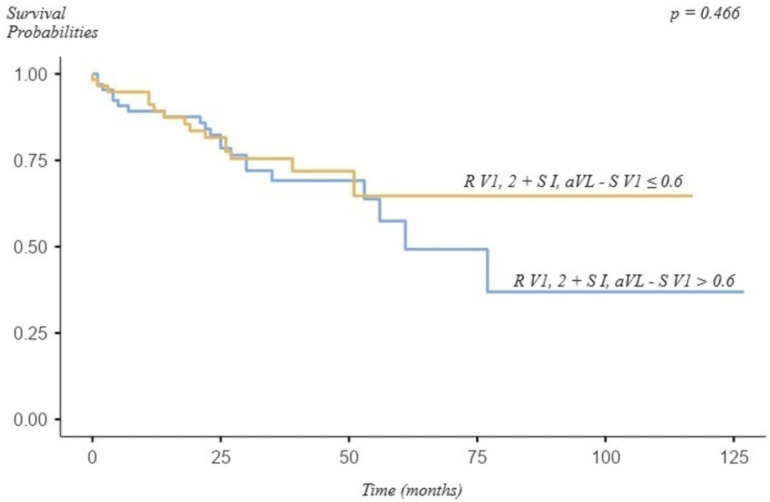
Survival of patients with pulmonary hypertension and “R V1, 2 + SI, aVL − S V1” > 0.6 and ≤ 0.6.

Even though patients who exceeded the defined cut-off (>0.6 mV) were more likely to be dead after more than 6 years, the difference was not statistically significant (*p* = 0.466; Figure 1).

In the univariate logistic regression analysis, exceedance of the 0.6 mV cut-off was a predictor for an mPAP > 20 mmHg (OR: 34.33; 95% CI: 10.53–111.95; *p* < 0.001) and a PVR > 5 WU or > 400 dyn∗sec∗cm^−5^ (OR: 17.14; 95% CI: 8.58–34.23; *p* < 0.001). In addition, the parameter was able to predict increased (NT-pro-) BNP levels (OR: 4.48; 95% CI: 1.59–12.60; *p* = 0.005) and patients with PAH categorised as high risk at the time of diagnosis (OR: 4.72; 95% CI: 1.24–17.93; *p* = 0.023). Exceedance of the 0.6 mV cut-off was not able to predict all-cause mortality, the need for long-term oxygen therapy (LTOT), or the 6MWD and NYHA classes (Table 4).

## 4. Discussion

### 4.1. Electrocardiographic Parameters for the Diagnosis of Pulmonary Hypertension

Previous studies describe a low sensitivity (range: 3–93%) and low NPV (range: 9–100%) but a relatively good specificity (range: 50–100%) and PPV (range: 0–100%) for ECG parameters for the diagnosis of PH or the detection of indirect PH signs, such as right atrial enlargement or RVH [6,9,12,13,14,15,16,17,18,19]. The diagnostic accuracy of electrocardiographic parameters was usually too low to safely diagnose or exclude PH [6,9,13,14,15,16,17,18]. However, the transferability of these results to the new mPAP threshold (>20 mmHg) is unclear as no study has investigated the diagnostic accuracy of ECG parameters applied with their current cut-offs for this threshold.

In the present study, most ECG parameters also showed a low sensitivity (range: 3–98%; mean: 39%) and NPV (range: 22–54%; mean: 42%) for the diagnosis of PH (mPAP > 20 mmHg) but a relatively high specificity (range: 3–100%; mean: 87%) and PPV (range: 60–100; mean: 90). This was even more evident in the EMPs (mean sensitivity: 40%; mean specificity: 96%; mean PPV: 95%; mean NPV: 45%). However, the diagnostic accuracy was inadequate in most of the ECG parameters. Some parameters, e.g., the parameters “QRS axis associated with right heart strain”, “R V1, V2 + S I, aVL − S V1 > 0.6 mV”, “R V1, V2 + S I, V6 − S V1 > 0.6 mV”, and “T wave inversion in V3”, exhibited relatively reliable sensitivity (60%, 52%, 53%, and 60%) and good specificity (93%, 97%, 97%, and 93%).

When previous studies applied new cut-offs, sensitivity (range: 21–100%) and the Youden index increased (range: 0.12–0.78), while specificity slightly decreased (range: 29–97%) [10,34,48,49,50,51,52]. However, again, the transferability of these results to the new mPAP threshold (>20 mmHg) is unclear as only one study has investigated the diagnostic accuracy of the new cut-offs of ECG parameters for this threshold [48].

Overall, in the present study, it was observed that the sensitivity (range: 3–98% to 29–81%; mean: 39% to 66%) and Youden index (range: −0.18–0.53 to −0.22–0.68; mean: 0.26 to 0.40) increased significantly when applying new cut-offs of ECG parameters for the diagnosis of PH (mPAP > 20 mmHg), but specificity (range: 3–100% to 37–92%; mean: 87% to 74%) slightly decreased. The parameters “(RI + SIII) – (SI + RIII)”, “R V1, V2 + S I, aVL − S V1”, and “R V1 + S V5, V6” and their new cut-offs (< −0.05 mV, > 0.12 mV, and > 0.51 mV) were able to diagnose PH (>20 mmHg) with good sensitivity (76%, 75%, and 69%) and specificity (92%, 80%, and 84%). Therefore, some ECG parameters and their optimised cut-offs could diagnose PH with a similar diagnostic accuracy as echocardiograms (sensitivity: 83–85%; specificity: 72–74%) [53,54].

In clinical practice, physicians either want to diagnose (such tests need high specificity and PPV) or exclude a specific disease (such tests need high sensitivity and NPV). Since the present study was able to show that ECG parameters for the diagnosis of PH only showed low sensitivity and NPV, the ECG alone seems unable to reliably rule out PH. Because ECG parameters have relatively high specificity and PPV, they could therefore theoretically diagnose PH. However, as they only showed low sensitivity, many patients with PH are missed by ECGs (high false negative rate). Therefore, the ECG alone is not able to reliably diagnose PH either. However, even with a lower mPAP threshold of >20 mmHg, the ECG can reveal the suspicion of PH, and it is therefore particularly important in primary care settings (with general practitioners and non-specialised cardiologists/pneumologists), and the application of the ECG should not be neglected. Physicians need to be aware that characteristic ECG findings suggest PH, which, in combination with typical symptoms (e.g., exertional dyspnoea) and laboratory results (elevated NT-pro-BNP levels), should result in a further diagnostic work-up. However, a completely normal ECG cannot rule out PH.

Even more optimal cut-offs of electrocardiographic parameters do not solve these problems and therefore cannot yet significantly improve the current PH diagnostic algorithm. Moreover, if the parameters’ cut-offs were adjusted to exhibit high sensitivity, the specificity suffered immensely (a high false positive rate and thus a lower PPV). However, if the ECG (e.g., CTEPH rule-out criteria) is combined with other methods such as clinical information (e.g., symptoms and CTEPH prediction score) and NT-pro-BNP values (e.g., CTEPH rule-out criteria), selected patients can be referred to echocardiography, and PH can be diagnosed or ruled out relatively well (sensitivity: 92%; specificity: 83%) [31]. This suggests that ECG findings should always be interpreted in the clinical context. Studies using artificial intelligence (AI) for PH diagnosis revealed promising results [55,56,57,58]. On this basis, there should be further studies to investigate the exact influence of electrocardiographic parameters in combination with AI on the current PH diagnostic algorithm and earlier PH diagnosis.

### 4.2. The Role of “R V1, V2 + S I, aVL − S V1” in Patients with Pulmonary Hypertension

Only few studies have investigated the use of “R V1, V2 + S I, aVL − S V1” for the detection of RVH and/or PH [8,9,10,11,18,19,35]. “R V1, V2 + S I, aVL − S V1” and the exceedance of a defined cut-off (0.6 mV) may be able to help diagnose PH or RVH, estimating pulmonary haemodynamics and indicating a successful therapeutic outcome [9,10,11,19,35].

Two studies concluded that a new cut-off (0.3 mV) was more suitable (sensitivity: 91–94%; specificity: 60–71%; Youden index: 0.51–0.65) [10,19]. However, in the present study, a different optimal cut-off (0.12 mV; sensitivity: 75%; specificity: 80%; Youden index: 0.55; AUC: 0.84; accuracy: 0.80) was determined.

Furthermore, exceedance of its commonly used cut-off (0.6 mV) was associated with statistically significantly more severe CTEPH (higher mPAP, PVR, and NT-pro-BNP levels and lower tricuspid annular plane systolic excursion (TAPSE)) and a statistically insignificantly increased risk of death and higher troponin levels in a previous study [8]. The present study largely confirms these findings and demonstrates that exceedance of the 0.6 mV cut-off of “R V1, V2 + S I, aVL − S V1” could be able to predict the diagnosis of (severe) PH, increased (NT-pro-) BNP levels, and high-risk category (only patients with PAH) at the time of diagnosis. However, the need for LTOT, worse 6MWD, and a higher NYHA class could not be predicted. Furthermore, this parameter was not able to predict all-cause mortality, and survival was not statistically significantly different, although patients who exceeded this cut-off were more likely to die (Figure 1).

Despite appearing interesting and being able to show a similar diagnostic accuracy as the echocardiogram for the diagnosis of PH with a new cut-off (0.12 mV; sensitivity and specificity: 75% and 80% vs. 83–85% and 72–74%), further, preferably large multi-centre studies are necessary to determine its exact role, especially in PH risk stratification [53,54].

### 4.3. Limitations

The present study stands out due to several of its features (a large PH sample size (*n* = 200), a large CG (*n* = 100) with invasively excluded PH, a new mPAP threshold of 20 mmHg applied, an extensive selection of analysed parameters, and a bicentric design). The fact that the CG consisted of patients with suspected CTEPH (most of them post pulmonary embolism) may have led to the CG not perfectly matching the general population. Therefore, the frequency of ECG signs typical for PH may have been overestimated (a false positive rate that was possibly too high) in the general population, and the specificity of ECG parameters could have been underestimated. However, this exact situation is present when a clinician faces a patient with persistent dyspnoea after pulmonary embolism and suspected CTEPH (real-world conditions). Nevertheless, the present study also has some limitations (due to it being a retrospective study, potential confounders could have been missed; no patients with group 2 or 3 PH were included; the referral centres for PAH and CTEPH with highly selected patients could have led to potential selection bias; inter-observer agreement was not tested; and there was no power calculation for sample size justification), all of which could decrease its external validity and complicate transferability to the general population of patients with PH. As PH expert centres usually receive referrals of patients with relatively high PH probability and therefore potentially haemodynamically more severe PH, overestimation of the ECG parameters’ prevalence/sensitivity (and, consecutively, of their PPV and NPV) cannot be ruled out completely. Furthermore, some of the ECG parameters applied in the present study may be too complex and time-consuming for clinical routine. Although most of the electrocardiographic parameters applied were already validated, their diagnostic accuracy was not validated internally for the new mPAP threshold of >20 mmHg in the present study [29].

## 5. Conclusions

The present study was able to create a reference work for the diagnostic accuracy of ECG parameters and their optimal cut-offs for the diagnosis of PH. Even the improved cut-offs’ electrocardiographic parameters alone are not able to reliably diagnose or rule out PH because of their low sensitivity. Therefore, the present findings are not able to significantly change the current PH diagnostic algorithm or the current PH guidelines. However, they might be helpful to reveal a suspicion of PH, especially in primary care settings (with general practitioners or non-specialised cardiologists/pneumologists), because some parameters demonstrated high specificity and PPVs. Moreover, some parameters with optimised cut-offs may potentially have a similar diagnostic accuracy as the echocardiogram. Combining ECG parameters with clinical information and other simple tests like NT-pro-BNP values is helpful to enhance the ability to reliably diagnose or exclude PH. The use of artificial intelligence may also be helpful for (earlier) PH diagnosis and should be investigated in further studies. The exceedance of a defined cut-off (0.6 mV) of the “R V1, V2 + S I, aVL − S V1” parameter was able to predict the diagnosis of (severe) PH but could not predict all-cause mortality, although patients exceeding the cut-off were more likely to die.

## Figures and Tables

**Table 1:** Patient data.

	Control Group	PAH	*p*-Value ^1^	CTEPH	*p*-Value ^2^
*n*	100	100	-	100	-
sex, m/f, *n*	45, 55 (45%, 55%)	43, 57 (43%, 57%)	0.887	48, 52 (48%, 52%)	0.777
age, years, median (IQR)	60.8 (17.0)	65.7 (18.7)	0.005	63.2 (17.8)	0.297
CTEPH, *n*	0 (0%)	0 (0%)	-	100 (100%)	-
PAH, *n*	0 (0%)	100 (100%)	-	0 (0%)	-
mPAP, mmHg, median (IQR)	16 (4)	47 (18)	<0.001	40 (13)	<0.001
PVR, dyn∗sec∗cm^−5^, median (IQR)	128 (69)	716 (409)	<0.001	536 (323)	<0.001
NT-pro-BNP, pg/mL, median (IQR)	-	-	-	515 (1264)	-
BNP, pg/mL, median (IQR)	-	273 (458)	-	-	-
6 min walking distance, m	-	288 (172)	-	407 (133)	-
NYHA I, *n*	-	0 (0%)	-	0 (0%)	-
NYHA II, *n*	-	13 (13%)	-	2 (2%)	-
NYHA III, *n*	-	81 (81%)	-	79 (79%)	-
NYHA IV, *n*	-	6 (6%)	-	19 (19%)	-
Low risk (three-strata model), *n*	-	7 (7%)	-	-	-
Intermediate risk (three-strata model), *n*	-	78 (78%)	-	-	-
High risk (three-strata model), *n*	-	15 (15%)	-	-	-

Annotations: ^1^ comparison of control group with PAH patients; ^2^ comparison of control group with CTEPH patients; BNP: brain natriuretic peptide; CTEPH: chronic thromboembolic pulmonary hypertension; IQR: interquartile range; mPAP: mean pulmonary arterial pressure; NYHA: New York Heart Association; PAH: pulmonary arterial hypertension; PH: pulmonary hypertension; PVR: pulmonary vascular resistance.

**Table 2:** Sensitivity and specificity of electrocardiographic main parameters for diagnosis of pulmonary hypertension.

	Sensitivity	Specificity	PPV	NPV
QRS axis associated with right heart strain, %	60	93	95	54
P wave amplitude in II ≥ 0.25 mV, %	26	99	98	40
P dextroatriale or P biatriale, %	42	99	99	46
Right or biventricular hypertrophy (SLI), %	45	92	92	46
qR pattern in V1, %	14	98	93	36
Right bundle branch block, %	35	89	86	41
R amplitude in V1 > 0.6 mV, %	18	100	100	38
S amplitude in V6 > 0.3 mV, %	41	95	94	45
R/S in V1 > 1.0, %	41	99	99	46
R V1, V2 + S I, aVL − S V1 > 0.6 mV, %	52	97	97	50
R V1, V2 + S I, V6 − S V1 > 0.6 mV, %	53	97	97	51
R V1 + S V5, V6 > 1.05 mV, %	32	99	99	42
Time to R peak in V1 (QRS < 120 ms) > 35 ms, %	61	85	89	52
Range, %	14–61	85–100	86–100	36–54
Average, %	40	96	95	45

Annotations: NPV: negative predictive value; PPV: positive predictive value; QRS axis associated with right heart strain: QRS axis > 90°, SIQIII type or SISIISIII type; SLI: Sokolow–Lyon index.

**Table 3:** Prediction of pulmonary hypertension—univariate and multivariate logistic regression.

	Univariate Logistic Regression			Multivariate Logistic Regression *		
	OR (95% CI)	*p*-Value	Nagelkerke’s *R*^2^	OR (95% CI)	*p*-Value	Nagelkerke’s *R*^2^
QRS axis associated with right heart strain	19.93 (8.79–45.18)	<0.001	0.36	6.10 (2.16–17.28)	<0.001	0.59
P wave amplitude in II ≥ 0.25 mV	33.80 (4.59–248.82)	<0.001	0.17			
P dextroatriale or biatriale	70.55 (9.63–516.58)	<0.001	0.31			
Right ventricular or biventricular hypertrophy (SLI)	9.41 (4.34–20.41)	<0.001	0.21			
qR pattern in V1	7.65 (1.78–32.85)	0.006	0.06			
Right bundle branch block	4.26 (2.14–8.50)	<0.001	0.09			
R amplitude in V1 > 0.6 mV	0.00 (0.00–∞)	0.980	0.14			
S amplitude in V6 > 0.3 mV	13.20 (5.14–33.88)	<0.001	0.22			
R/S in V1 > 1.0	65.85 (9.00–482.04)	<0.001	0.30			
R V1, V2 + S I, aVL − S V1 > 0.6 mV	34.33 (10.53–111.95)	<0.001	0.35			
R V1, V2 + S I, V6 − S V1 > 0.6 mV	35.74 (10.96–116.53)	<0.001	0.35			
R V1 + S V5, V6 > 1.05 mV	46.59 (6.36–341.32)	<0.001	0.22			
Time to R peak in V1 (QRS < 120 ms) > 35 ms	8.51 (4.46–16.21)	<0.001	0.25	3.69 (1.45–9.38)	0.006	0.59

Annotation: *: all parameters were included in the multivariate logistic regression analysis, but only statistically significant results are displayed. OR: odds ratio; QRS axis associated with right heart strain: QRS axis > 90°, SIQIII type or SISIISIII type; SLI: Sokolow–Lyon index.

**Table 4:** “R V1, 2 + SI, aVL − S V1” for the prediction of (severe) pulmonary hypertension and consecutive mortality—univariate logistic regression.

Parameter	OR (95% CI)	*p*-Value	Nagelkerke’s *R*^2^
Pulmonary hypertension	34.33 (10.53–111.95)	<0.001	0.35
Severe pulmonary hypertension	17.14 (8.58–34.23)	<0.001	0.37
All-cause death	1.49 (0.71–3.11)	0.290	0.01
Elevated (NT-pro-) BNP	4.48 (1.59–12.60)	0.005	0.09
Need for LTOT	1.39 (0.75–2.59)	0.294	0.01
6MWD < 165 m	7.30 (0.89–60.00)	0.064	0.10
6MWD = 165–440 m	0.97 (0.48–1.95)	0.932	0.00
6MWD > 440 m	0.67 (0.32–1.40)	0.287	0.01
NYHA II	1.08 (0.38–3.11)	0.883	0.00
NYHA III	0.95 (0.48–1.90)	0.887	0.00
NYHA IV	1.02 (0.44–2.37)	0.957	0.00
Low risk ^#^	0.14 (0.02–1.24)	0.077	0.11
Intermediate risk ^#^	0.66 (0.25–1.72)	0.392	0.01
High risk ^#^	4.72 (1.24–17.93)	0.023	0.11

Annotations: ^#^ only patients with PAH; 6MWD: six-minute walk distance; BNP: brain natriuretic peptide; LTOT: long-term O_2_ therapy; NYHA: New York Heart Association; OR: odds ratio; severe pulmonary hypertension: PVR > 5 WU/400 dyn∗sec∗cm^−5^.

## Data Availability

The original contributions presented in this study are included in the article and Supplementary Materials. Further inquiries can be directed to the corresponding author.

## Supplementary Materials

The following supporting information can be downloaded at https://www.mdpi.com/article/10.3390/jcm13247613/s1, Table S1: Patient data, Table S2: Main electrocardiographic data, Table S3: Electrocardiographic data, Table S4: The sensitivity and specificity of electrocardiographic parameters for the diagnosis of pulmonary arterial, chronic thromboembolic, and severe and non-severe pulmonary hypertension, Table S5: Optimal cut-offs of electrocardiographic parameters for the diagnosis of pulmonary hypertension, Table S6: Patient characteristics and electrocardiographic data of patients with “R V1, V2 + SI, aVL − S V1” > 0.6 and ≤ 0.6 mV in pulmonary hypertension, Table S7: Definitions of and annotations on complex electrocardiographic parameters.
