# Supplementary material for: The Diagnostic Accuracy of an Electrocardiogram in Pulmonary Hypertension and the Role of “R V1, V2 + S I, aVL − S V1”
Source: J Clin Med. 2024 Dec 13;13(24):7613. doi: 10.3390/jcm13247613 (PMC11679519; doi:10.3390/jcm13247613)
Supplement: Supplementary file 1 [file jcm-13-07613-s001.zip › jcm-3347218-supplementary.pdf]

Table S1. Patient data.

	Control group	PAH	*p*-value[1]	CTEPH	*p*-value[2]	Non-severe PH	Severe PH	*p*-value[3]
n	100	100	-	100	-	40 (20%)	160 (80%)	-
sex, m/f, *n*	45, 55 (45%, 55%)	43, 57 (43%, 57%)	0.887	48, 52 (48%, 52%)	0.777	17, 23 (42.5%, 57.5%)	74, 86 (46%, 54%)	0.725
age, years, median (IQR)	60.8 (17.0)	65.7 (18.7)	0.005	63.2 (17.8)	0.297	67.3 (17.0)	63.0 (18.6)	0.137
CTEPH, *n*	0 (0%)	0 (0%)	-	100 (100%)	-	27 (68%)	73 (46%)	0.021
PAH, *n*	0 (0%)	100 (100%)	-	0 (0%)	-	13 (32%)	87 (54%)	0.021
IPAH, *n*	-	73 (73%)	-	-	-	13 (100%)[#]	60 (69%)[#]	0.018
PAH-CTD, *n*	-	15 (15%)	-	-	-	0 (0%)[#]	15 (17%)[#]	0.207
PoPH, *n*	-	11 (11%)	-	-	-	0 (0%)[#]	11 (13%)[#]	0.350
PAH-HIV, *n*	-	1 (1%)	-	-	-	0 (0%)[#]	1 (1%)[#]	1.000
RAP, mm Hg, median (IQR)	-	9 (5)	-	6 (4)	-	7 (3)	8 (5)	0.008
mPAP, mm Hg, median (IQR)	16 (4)	47 (18)	< 0.001	40 (13)	< 0.001	31 (6)	47 (13)	< 0.001
CI, L/min/m^2, median (IQR)	2.5 (0.5)	2.2 (0.7)	< 0.001	2.6 (0.8)	0.091	2.8 (0.6)	2.3 (0.7)	< 0.001
CO, L/min, median (IQR)	5 (1.6)	3.7 (1.1)	< 0.001	4.7 (1.5)	0.266	5.4 (1.8)	4.0 (1.2)	< 0.001
PVR, dyn∗sec∗cm^{-5}, median (IQR)	128 (69)	716 (409)	< 0.001	536 (323)	< 0.001	290 (82)	677 (316)	< 0.001
NT-pro-BNP, pg/ml, median (IQR)	-	-	-	515 (1264)	-	142 (124)[+]	1120 (1521)[+]	< 0.001

BNP, pg/ml, median (IQR)	-	273 (458)	-	-	-	69 (159)$^{\#}$	333 (486)$^{\#}$	< 0.001
6 minute walk distance, m	-	288 (172)	-	407 (133)	-	436 (153)	365 (157)	0.020
NYHA I, *n*	-	0 (0%)	-	0 (0%)	-	0 (0%)	0 (0%)	-
NYHA II, *n*	-	13 (13%)	-	2 (2%)	-	5 (12.5%)	10 (6%)	0.187
NYHA III, *n*	-	81 (81%)	-	79 (79%)	-	31 (77.5%)	129 (81%)	0.662
NYHA IV, *n*	-	6 (6%)	-	19 (19%)	-	4 (10%)	21 (13%)	0.791
Low risk (three-strata model), *n*	-	7 (7%)	-	-	-	6 (46%)	1 (1%)	< 0.001
Intermediate risk (three-strata model) , *n*	-	78 (78%)	-	-	-	7 (54%)	71 (82%)	0.035
High risk (three-strata model) , *n*	-	15 (15%)	-	-	-	0 (0%)	15 (17%)	0.207
BPA, *n*	-	-	-	100 (100%)	-	27 (100%)$^{+}$	73 (100%)$^{+}$	1.000
Initial pharmacological monotherapy, *n*	-	65 (65%)	-	-	-	10 (77%)$^{\#}$	55 (63%)$^{\#}$	0.028
Initial pharmacological dual therapy, *n*	-	31 (31%)	-	-	-	0 (0%)$^{\#}$	31 (36%)$^{\#}$	0.028
Initial pharmacological triple therapy, *n*	-	1 (1%)	-	-	-	0 (0%)$^{\#}$	1 (1%)$^{\#}$	1.000
LTOT, *n* (median dosage)	-	43, 43% (2)	-	18, 23% (2)	-	8, 24% (2)	53, 36% (2)	0.323 (0.388)

Annotation: $^{+}$: only CTEPH patients, $^{\#}$: only PAH patients, ^{1}: comparison of control group with PAH patients, ^{2}: comparison of control group with CTEPH patients, ^{3}: comparison of patients with non-severe and severe pulmonary hypertension, BNP: brain natriuretic peptide, BPA: balloon pulmonary angioplasty, CI: cardiac index, CO: cardiac output, CTEPH: chronic thromboembolic pulmonary hypertension, IPAH: idiopathic pulmonary arterial hypertension, IQR: interquartile range, LTOT: long-term O_2 therapy, mPAP: mean pulmonary arterial pressure, non-severe PH: PVR ≤ 5 WU/400 $dyn*sec*cm^{-5}$, NYHA: New York Heart Association, PAH: pulmonary arterial hypertension, PAH-CTD: PAH associated with connective tissue disease, PAH-HIV: pulmonary arterial hypertension associated with an HIV infection, PH: pulmonary hypertension, PoPH: portopulmonary hypertension, PVR: pulmonary vascular resistance, RAP: right atrial pressure, severe PH: PVR > 5 WU/400 $dyn*sec*cm^{-5}$

Table S2. Main electrocardiographic data.

	Control group	PAH	*p*-value[1]	CTEPH	*p*-value[2]	Non-severe PH	Severe PH	*p*-value[3]
QRS axis associated with right heart strain, *n* (%)	7 (7%)	63 (63%)	< 0.001*	57 (57%)	< 0.001*	14 (35%)	106 (66%)	< 0.001*
P wave amplitude in II, mV, median (IQR)	0.12 (0,07)	0.18 (0.09)	< 0.001*	0.2 (0.1)	< 0.001*	0.20 (0.10)	0.20 (0.15)	0.004
P wave amplitude in II ≥ 0.25 mV, *n* (%)	1 (1%)	16 (17%)	< 0.001*	34 (34%)	< 0.001*	4 (11%)	46 (30%)	0.014
P dextroatriale or biatriale, *n* (%)	1 (1%)	34 (37%)	< 0.001*	46 (47%)	< 0.001*	6 (15%)	74 (46%)	< 0.001*
Right ventricular or biventricular hypertrophy (SLI), *n* (%)	8 (8%)	44 (44%)	< 0.001*	46 (46%)	< 0.001*	11 (28%)	79 (49%)	0.013
qR pattern in V1, *n* (%)	2 (2%)	13 (13%)	0.005	14 (14%)	0.003*	0 (0%)	27 (17%)	0.003*
Right bundle branch block, *n* (%)	11 (11%)	39 (39%)	< 0.001*	30 (30%)	0.001*	10 (25%)	59 (37%)	0.194
R amplitude in V1, mV, median (IQR)	0.1 (0.1)	0.26 (0.41)	< 0.001*	0.3 (0.35)	< 0.001*	0.15 (0.15)	0.31 (0.39)	< 0.001*
R amplitude in V1 > 0.6 mV, *n* (%)	0 (0%)	22 (22%)	< 0.001*	13 (13%)	< 0.001*	2 (5%)	33 (21%)	0.019
S amplitude in V6, mV, median (IQR)	0.05 (0.15)	0.24 (0.29)	< 0.001*	0.25 (0.4)	< 0.001*	0.10 (0.20)	0.30 (0.35)	< 0.001*
S amplitude in V6 > 0.3 mV, *n* (%)	5 (5%)	39 (39%)	< 0.001*	43 (43%)	< 0.001*	5 (13%)	77 (48%)	< 0.001*

R/S in V1, median (IQR)	0.16 (0.22)	0.91 (1.99)	< 0.001*	1 (2.3)	< 0.001*	0.30 (0.48)	1.25 (2.50)	< 0.001*
R/S in V1 > 1.0, *n* (%)	1 (1%)	34 (41%)	< 0.001*	40 (40%)	< 0.001*	4 (10%)	70 (49%)	< 0.001*
R V1, V2 + S I, aVL - S V1, mV, median (IQR)	-0.25 (0.58)	0.64 (1.03)	< 0.001*	0.70 (0.75)	< 0.001*	0.30 (0.50)	0.78 (0.80)	< 0.001*
R V1, V2 + S I, aVL - S V1 > 0.6 mV, *n* (%)	3 (3%)	51 (51%)	< 0.001*	52 (52%)	< 0.001*	8 (20%)	95 (59%)	< 0.001*
R V1, V2 + S I, V6 - S V1, mV, median (IQR)	-0.23 (0.65)	0.66 (0.98)	< 0.001*	0.7 (0.9)	< 0.001*	0.15 (0.60)	0.77 (0.87)	< 0.001*
R V1, V2 + S I, V6 - S V1 > 0.6 mV, *n* (%)	3 (3%)	53 (53%)	< 0.001*	52 (52%)	< 0.001*	9 (23%)	96 (60%)	< 0.001*
R V1 + S V5, V6, mV, median (IQR)	0.30 (0.25)	0.76 (0.60)	< 0.001*	0.8 (0.78)	< 0.001*	0.50 (0.38)	0.86 (0.73)	< 0.001*
R V1 + S V5, V6 > 1.05 mV, *n* (%)	1 (1%)	30 (30%)	< 0.001*	34 (34%)	< 0.001*	1 (3%)	63 (39%)	< 0.001*
Time to R peak in V1 (QRS < 120 ms), ms, median (IQR)	20 (8)	55 (42)	< 0.001*	50 (31)	< 0.001*	25 (25)	55 (25)	< 0.001*
Time to R peak in V1 (QRS < 120 ms) > 35 ms, *n* (%)	14 (15%)	47 (64%)	< 0.001*	57 (57%)	< 0.001*	10 (28%)	94 (69%)	< 0.001*

Annotation: [1]: comparison of control group with PAH patients, [2]: comparison of control group with CTEPH patients, [3]: comparison of patients with non-severe and severe pulmonary hypertension, *: a *p*-value < 0.004 was considered statistically significant after Bonferroni correction, CTEPH: chronic thromboembolic pulmonary hypertension, IQR: interquartile range, non-severe PH: PVR ≤ 5 WU/400 dyn∗sec∗cm^{-5}, PAH: pulmonary arterial hypertension, PH: pulmonary hypertension, QRS axis associated with right heart strain: QRS axis > 90°, SIQIII type or SISIISIII type, SLI: Sokolow-Lyon index, severe PH: PVR > 5 WU/400 dyn∗sec∗cm^{-5}

Table S3. Electrocardiographic data.

	Control group	PAH	p-value[1]	CTEPH	p-value[2]	Non-severe PH	Severe PH	p-value[3]
Sinus rhythm, *n* (%)	98 (98%)	91 (91%)	0.058	99 (99%)	1.000	38 (95%)	152 (95%)	1.000
Heart rate, bpm, median (IQR)	75 (21)	74 (17)	0.695	84 (22)	< 0.001	75 (16)	81 (17)	0.070
QRS axis > 90°, *n* (%)	3 (3%)	39 (39%)	< 0.001	42 (42%)	< 0.001	6 (15%)	75 (47%)	< 0.001
QRS axis > 120°, *n* (%)	0 (0%)	9 (9%)	0.003	16 (16%)	< 0.001	1 (3%)	24 (15%)	0.032
SISIISIII type, *n* (%)	4 (4%)	13 (13%)	0.040	7 (7%)	0.537	2 (5%)	18 (11%)	0.377
SIQIII type, *n* (%)	0 (0%)	11 (11%)	< 0.001	8 (8%)	0.007	6 (15%)	13 (8%)	0.225
QRS axis associated with right heart strain, *n* (%)	7 (7%)	63 (63%)	< 0.001*	57 (57%)	< 0.001*	14 (35%)	106 (66%)	< 0.001*
Left axis deviation, *n* (%)	38 (38%)	17 (17%)	0.001	6 (6%)	< 0.001	5 (13%)	18 (11%)	0.786
Normal QRS axis, *n* (%)	55 (55%)	20 (20%)	< 0.001	36 (36%)	0.010	21 (53%)	35 (22%)	< 0.001
P wave amplitude in II, mV, median (IQR)	0.12 (0,07)	0.18 (0.09)	< 0.001*	0.2 (0.1)	< 0.001*	0.20 (0.10)	0.20 (0.15)	0.004
P wave amplitude in II ≥ 0.25 mV, *n* (%)	1 (1%)	16 (17%)	< 0.001*	34 (34%)	< 0.001*	4 (11%)	46 (30%)	0.014
Highest P wave, mV, median (IQR)	0.12 (0.07)	0.2 (0.07)	< 0.001	0.2 (0.1)	< 0.001	0.20 (0.08)	0.20 (0.13)	< 0.001
P dextroatriale, *n* (%)	1 (1%)	17 (19%)	< 0.001	40 (41%)	< 0.001	6 (16%)	51 (34%)	0.046
P biatriale, *n* (%)	0 (0%)	17 (19%)	< 0.001	6 (6%)	< 0.001	0 (0%)	23 (15%)	0.005

P dextroatriale or biatriale, *n* (%)	1 (1%)	34 (37%)	< 0.001*	46 (47%)	< 0.001*	6 (15%)	74 (46%)	< 0.001*
Longest P wave, ms, median (IQR)	120 (19)	130 (20)	0.022	120 (0)	0.020	120 (10)	120 (20)	0.351
Shortest P wave, ms, median (IQR)	80 (20)	80 (20)	0.028	80 (10)	0.032	80 (24)	80 (20)	0.243
P wave dispersion, ms, median (IQR)	40 (20)	40 (20)	0.891	40 (10)	0.907	40 (19)	40 (10)	0.653
PQ interval, ms, median (IQR)	150 (26)	170 (45)	< 0.001	150 (30)	0.333	150 (30)	155 (40)	0.585
PR interval, ms, median (IQR)	150 (26)	170 (51)	0.165	160 (23)	0.476	180 (40)	160 (38)	0.375
Supraventricular extrasystoles, *n* (%)	4 (4%)	7 (7%)	0.537	5 (5%)	1.000	5 (13%)	7 (4%)	0.066
Right ventricular hypertrophy (SLI), *n* (%)	8 (8%)	43 (43%)	< 0.001	46 (46%)	< 0.001	11 (28%)	78 (49%)	0.020
Biventricular hypertrophy (SLI), *n* (%)	0 (0%)	1 (1%)	1.000	0 (0%)	-	0 (0%)	1 (1%)	1.000
Right ventricular or biventricular hypertrophy (SLI), *n* (%)	8 (8%)	44 (44%)	< 0.001*	46 (46%)	< 0.001*	11 (28%)	79 (49%)	0.013
qR pattern in V1, *n* (%)	2 (2%)	13 (13%)	0.005	14 (14%)	0.003*	0 (0%)	27 (17%)	0.003*
Right bundle branch block, *n* (%)	11 (11%)	39 (39%)	< 0.001*	30 (30%)	0.001*	10 (25%)	59 (37%)	0.194
Complete right bundle branch block, *n* (%)	6 (6%)	26 (26%)	< 0.001	13 (13%)	0.146	5 (13%)	34 (21%)	0.268
Incomplete right bundle branch block, *n* (%)	5 (5%)	13 (13%)	0.081	17 (17%)	0.011	5 (13%)	25 (16%)	0.805

QRS interval, ms, median	95 (11)	100 (30)	< 0.001	95 (20)	0.691	100 (16)	100 (20)	0.571
R amplitude in I, mV, median (IQR)	0.60 (0.40)	0.40 (0.31)	< 0.001	0.3 (0.3)	< 0.001	0.50 (0.36)	0.33 (0.30)	0.008
R amplitude in I ≤ 0.2 mV, n (%)	7 (7%)	79 (79%)	< 0.001	35 (35%)	< 0.001	20 (50%)	94 (59%)	0.373
R amplitude in III, mV, median (IQR)	0.25 (0.30)	0.48 (0.56)	< 0.001	0.8 (0.6)	< 0.001	0.56 (0.48)	0.62 (0.67)	0.323
R amplitude in aVR, mV, median (IQR)	0.6 (0.37)	0.41 (0.30)	< 0.001	0.5 (0.3)	0.067	0.55 (0.29)	0.43 (0.30)	< 0.001
R amplitude in aVR > 0.4 mV, *n* (%)	76 (76%)	50 (50%)	< 0.001	65 (65%)	0.121	30 (75%)	85 (53%)	0.013
R amplitude in V1, mV, median (IQR)	0.1 (0.1)	0.26 (0.41)	< 0.001*	0.3 (0.35)	< 0.001*	0.15 (0.15)	0.31 (0.39)	< 0.001*
R amplitude in V1 > 0.6 mV, *n* (%)	0 (0%)	22 (22%)	< 0.001*	13 (13%)	< 0.001*	2 (5%)	33 (21%)	0.019
R-amplitude in V2, mV, median (IQR)	0.2 (0.25)	0.38 (0.48)	< 0.001	0.3 (0.4)	< 0.001	0.30 (0.32)	0.35 (0.40)	0.505
R amplitude in V6, mV, median (IQR)	0.75 (0.35)	0.60 (0.36)	< 0.001	0.8 (0.5)	0.107	0.70 (0.35)	0.70 (0.49)	0.126
R amplitude in V6 < 0.3 mV, *n* (%)	4 (4%)	11 (11%)	0.105	1 (1%)	0.369	0 (0%)	12 (8%)	0.129
S amplitude in I, mV, median (IQR)	0.05 (0.1)	0.33 (0.36)	< 0.001	0.35 (0.4)	< 0.001	0.20 (0.21)	0.36 (0.36)	< 0.001
S amplitude in III, mV, median (IQR)	0.15 (0.40)	0.10 (0.27)	0.143	0.13 (0.3)	0.361	0.03 (0.25)	0.10 (0.30)	0.227
S amplitude in aVL, mV, median (IQR)	0 (0.1)	0.36 (0.36)	< 0.001	0.5 (0.4)	< 0.001	0.33 (0.27)	0.45 (0.51)	0.025

S amplitude in V1, mV, median (IQR)	0.55 (0.35)	0.25 (0.40)	< 0.001	0.3 (0.33)	< 0.001	0.49 (0.30)	0.23 (0.30)	< 0.001
S amplitude in V1 < 0.2 mV, *n* (%)	7 (7%)	35 (35%)	< 0.001	30 (30%)	< 0.001	4 (10%)	61 (38%)	< 0.001
S amplitude in V5, mV, median (IQR)	0.2 (0.25)	0.39 (0.34)	< 0.001	0.5 (0.51)	< 0.001	0.27 (0.28)	0.48 (0.47)	< 0.001
S amplitude in V5 > 1.0 mV, *n* (%)	0 (0%)	9 (9%)	0.003	10 (10%)	0.002	1 (3%)	18 (11%)	0.131
S amplitude in V6, mV, median (IQR)	0.05 (0.15)	0.24 (0.29)	< 0.001*	0.25 (0.4)	< 0.001*	0.10 (0.20)	0.30 (0.35)	< 0.001*
S amplitude in V6 > 0.3 mV, *n* (%)	5 (5%)	39 (39%)	< 0.001*	43 (43%)	< 0.001*	5 (13%)	77 (48%)	< 0.001*
R/S in V1, median (IQR)	0.16 (0.22)	0.91 (1.99)	< 0.001*	1 (2.3)	< 0.001*	0.30 (0.48)	1.25 (2.50)	< 0.001*
R/S in V1 > 1.0, *n* (%)	1 (1%)	34 (41%)	< 0.001*	40 (40%)	< 0.001*	4 (10%)	70 (49%)	< 0.001*
R/S in V5, median (IQR)	2.76 (4.64)	1.45 (1.68)	< 0.001	1.89 (2.41)	< 0.001	2.50 (2.17)	1.39 (1.69)	< 0.001
R/S in V5 < 0.75, *n* (%)	4 (5%)	15 (16%)	0.015	10 (10%)	0.176	1 (3%)	24 (15%)	0.054
R/S in V6, median (IQR)	5.90 (6.54)	1.75 (2.04)	< 0.001	2.67 (3.15)	< 0.001	4.00 (4.05)	1.78 (2.17)	< 0.001
R/S in V6 < 0.4, *n* (%)	1 (2%)	3 (4%)	0.637	1 (1%)	1.000	0 (0%)	4 (3%)	1.000
S > R or S > 40 ms in I, II or III, *n* (%)	50 (50%)	78 (78%)	< 0.001	68 (68%)	0.014	18 (45%)	128 (80%)	< 0.001
S > R or S > 40 ms in V6, *n* (%)	8 (8%)	43 (43%)	< 0.001	21 (24%)	0.002	4 (13%)	60 (39%)	0.006
R/S V1 > R/S in V3 or V4, *n* (%)	3 (3%)	34 (42%)	< 0.001	25 (29%)	< 0.001	3 (8%)	56 (42%)	< 0.001

R/S in V5 : R/S in V1, median (IQR)	18 (43.7)	1.54 (6.54)	< 0.001	1.94 (7.95)	< 0.001	6.38 (14.90)	1.13 (3.24)	< 0.001
R/S V5: R/S V1 < 0.04, *n* (%)	1 (1%)	4 (5%)	0.197	0 (0%)	0.459	0 (0%)	4 (3%)	0.582
(RI + SIII) - (SI + RIII), mV, median (IQR)	0.4 (0.86)	-0.39 (0.94)	< 0.001	-0.65 (0.9)	< 0.001	-0.33 (0.79)	-0.63 (0.94)	0.009
(RI + SIII) − (SI + RIII) < 1.5 mV, *n* (%)	97 (97%)	98 (98%)	1.000	98 (98%)	1.000	38 (95%)	158 (99%)	0.179
R V1, V2 + S I, aVL - S V1, mV, median (IQR)	-0.25 (0.58)	0.64 (1.03)	< 0.001*	0.70 (0.75)	< 0.001*	0.30 (0.50)	0.78 (0.80)	< 0.001*
R V1, V2 + S I, aVL - S V1 > 0.6 mV, *n* (%)	3 (3%)	51 (51%)	< 0.001*	52 (52%)	< 0.001*	8 (20%)	95 (59%)	< 0.001*
R V1, V2 + S I, V6 - S V1, mV, median (IQR)	-0.23 (0.65)	0.66 (0.98)	< 0.001*	0.7 (0.9)	< 0.001*	0.15 (0.60)	0.77 (0.87)	< 0.001*
R V1, V2 + S I, V6 - S V1 > 0.6 mV, *n* (%)	3 (3%)	53 (53%)	< 0.001*	52 (52%)	< 0.001*	9 (23%)	96 (60%)	< 0.001*
R V1 + S V5, V6, mV, median (IQR)	0.30 (0.25)	0.76 (0.60)	< 0.001*	0.8 (0.78)	< 0.001*	0.50 (0.38)	0.86 (0.73)	< 0.001*
R V1 + S V5, V6 > 1.05 mV, *n* (%)	1 (1%)	30 (30%)	< 0.001*	34 (34%)	< 0.001*	1 (3%)	63 (39%)	< 0.001*
Time to R peak in V1 (QRS < 120 ms), ms, median (IQR)	20 (8)	55 (42)	< 0.001*	50 (31)	< 0.001*	25 (25)	55 (25)	< 0.001*

Time to R peak in V1 (QRS < 120 ms) > 35 ms, *n* (%)	14 (15%)	47 (64%)	< 0.001*	57 (57%)	< 0.001*	10 (28%)	94 (69%)	< 0.001*
Ventricular extrasystoles, *n* (%)	2 (2%)	6 (6%)	0.279	3 (3%)	1.000	1 (3%)	8 (5%)	0.691
T wave inversion in II, *n* (%)	1 (1%)	35 (35%)	< 0.001	26 (27%)	< 0.001	4 (10%)	57 (36%)	0.002
T wave inversion in III, *n* (%)	14 (18%)	46 (47%)	< 0.001	45 (46%)	< 0.001	10 (26%)	81 (51%)	0.004
T wave inversion in aVF, *n* (%)	3 (3%)	39 (39%)	< 0.001	36 (36%)	< 0.001	8 (20%)	67 (42%)	0.011
T wave inversion in V1, *n* (%)	74 (85%)	79 (81%)	0.444	84 (93%)	0.091	29 (76%)	134 (89%)	0.057
T wave inversion in V2, *n* (%)	11 (12%)	59 (60%)	< 0.001	52 (58%)	< 0.001	15 (39%)	96 (64%)	0.006
T wave inversion in V3, *n* (%)	6 (7%)	60 (60%)	< 0.001	58 (60%)	< 0.001	9 (23%)	109 (69%)	< 0.001
Longest T wave, ms, median (IQR)	260 (40)	340 (90)	< 0.001	300 (70)	< 0.001	300 (40)	320 (80)	0.081
Shortest T wave, ms, median (IQR)	165 (40)	210 (50)	< 0.001	150 (40)	< 0.001	170 (60)	180 (70)	0.237
T wave dispersion, ms, median (IQR)	80 (40)	120 (80)	< 0.001	140 (60)	< 0.001	120 (60)	140 (80)	0.318
QT interval, ms, median (IQR)	370 (60)	440 (80)	< 0.001	390 (60)	< 0.001	400 (40)	420 (100)	0.042
QTc interval (Bazett), ms, median (IQR)	409 (41)	493 (94)	< 0.001	449 (76)	< 0.001	445 (39)	483 (104)	< 0.001
QTc interval (Fridericia), ms, median (IQR)	398 (33)	474 (96)	< 0.001	425 (68)	< 0.001	426 (36)	460 (100)	0.003
QTc interval (Hodges), ms, median (IQR)	397 (31)	468 (89)	< 0.001	424 (60)	< 0.001	424 (32)	460 (94)	0.001

QTc interval (Framingham), ms, median (IQR)	398 (30)	468 (89)	< 0.001	390 (60)	0.142	400 (55)	440 (115)	0.008

Annotation: [1]: comparison of control group with PAH patients, [2]: comparison of control group with CTEPH patients, [3]: comparison of patients with non-severe and severe pulmonary hypertension, *: a *p*-value < 0.004 was considered statistically significant after Bonferroni correction, bpm: beats per minute, CTEPH: chronic thromboembolic pulmonary hypertension, IQR: interquartile range, left axis deviation: < 30°, normal QRS axis: 30-90°, non-severe PH: PVR ≤ 5 WU/400 dyn∗sec∗cm^{-5}, PAH: pulmonary arterial hypertension, PH: pulmonary hypertension, PQ/PR interval: PR interval was only measured when no Q was detectable, QRS axis associated with right heart strain: QRS axis > 90°, SIQIII type or SISIISIII type, SLI: Sokolow-Lyon index, severe PH: PVR > 5 WU/400 dyn∗sec∗cm^{-5}

Table S4. Sensitivity and specificity of electrocardiographic parameters for the diagnosis of pulmonary arterial, chronic thromboembolic, severe and non-severe pulmonary hypertension.

	PAH				CTEPH				Non-severe PH				Severe PH			
	Sens.	Spec.	PPV	NPV	Sens.	Spec.	PPV	NPV	Sens.	Spec.	PPV	NPV	Sens.	Spec.	PPV	NPV
QRS axis > 90°, %	39	97	93	61	42	97	93	63	15	97	67	74	47	97	96	53
QRS axis > 120°, %	9	100	100	52	16	100	100	54	3	100	100	72	15	100	100	42
SISIISIII type, %	13	96	77	53	7	96	64	51	5	96	33	72	11	96	82	40
SIQIII type, %	11	100	100	53	8	100	100	52	15	100	100	75	8	100	100	41
QRS axis associated with right heart strain, %	63	93	90	72	57	93	89	68	35	93	67	78	66	93	94	63
P wave amplitude in II ≥ 0.25 mV, %	17	99	94	54	34	99	97	60	11	99	80	73	30	99	98	47
P dextroatriale or P biatriale, %	37	99	97	61	47	99	98	65	15	99	86	74	46	99	99	54
Right or biventricular hypertrophy (SLI), %	44	92	85	62	46	92	85	63	28	92	58	76	49	92	91	53
qR pattern in V1, %	13	98	87	53	14	98	88	53	0	98	0	50	17	98	93	42
Right bundle branch block, %	39	89	78	59	30	89	73	56	25	89	48	75	37	89	84	47
R amplitude in I ≤ 0.2 mV, %	79	93	92	82	35	93	83	59	50	93	74	82	59	93	93	59
R amplitude in aVR > 0.4 mV, %	50	24	40	32	65	24	46	41	75	24	28	71	53	24	53	24
R amplitude in V1 > 0.6 mV, %	22	100	100	56	13	100	100	54	5	100	100	73	21	100	44	31

R amplitude in V6 < 0.3 mV, %	11	96	73	52	1	96	20	49	0	96	0	71	8	96	75	39
S amplitude in V1 < 0.2 mV, %	35	93	83	59	30	93	81	57	10	93	36	72	38	93	90	48
S amplitude in V5 > 1.0 mV, %	9	100	100	52	10	100	100	53	3	100	100	72	11	100	100	41
S amplitude in V6 > 0.3 mV, %	39	95	89	61	43	95	90	63	13	95	50	73	48	95	94	53
R/S in V1 > 1.0, %	41	99	98	63	40	99	98	62	10	99	80	73	49	99	99	55
R/S in V5 < 0.75, %	16	95	76	53	10	95	67	51	3	95	17	71	15	95	83	41
R/S in V6 < 0.4, %	4	98	67	51	1	98	33	50	0	98	0	71	3	98	71	39
S > R or S > 40 ms in I, II or III, %	78	50	61	69	68	50	58	61	45	50	27	69	80	50	72	61
S > R or S > 40 ms in V6, %	43	92	84	62	24	92	75	55	13	92	39	72	39	92	89	48
R/S V1 > R/S in V3 or V4, %	42	97	93	63	29	97	91	58	8	97	50	72	42	97	96	51
R/S V5: R/S V1 < 0.04, %	5	99	83	51	0	99	0	50	0	99	0	71	3	99	83	39
RI + SIII − SI + RIII < 1.5 mV, %	98	3	50	60	98	3	50	60	95	3	28	60	99	3	62	60
R V1, V2 + S I, aVL - S V1 > 0.6 mV, %	51	97	94	66	52	97	95	67	20	97	73	75	59	97	97	60
R V1, V2 + S I, V6 - S V1 > 0.6 mV, %	53	97	95	67	52	97	95	67	23	97	75	76	60	97	97	60
R V1 + S V5, V6 > 1.05 mV, %	30	99	97	59	34	99	97	60	3	99	50	72	39	99	98	50
Time to R peak in V1 (QRS < 120 ms) > 35 ms, %	64	85	81	70	57	85	79	66	28	85	42	75	69	85	88	63
T wave inversion in II, %	35	99	97	60	27	99	96	58	10	99	80	73	36	99	98	49

T wave inversion in III, %	47	82	72	61	46	82	72	60	26	82	46	73	51	82	87	51
T wave inversion in aVF, %	39	97	93	61	36	97	92	60	20	97	73	75	42	97	96	51
T wave inversion inV1, %	81	15	49	44	93	15	52	68	76	15	26	60	89	15	63	46
T wave inversion in V2, %	60	88	83	69	58	88	83	68	39	88	56	78	64	88	90	60
T wave inversion in V3, %	60	93	90	70	60	93	90	70	23	93	56	75	69	93	94	65
Range, %	4-98	3-100	40-100	32-82	0-98	3-100	0-100	41-70	0-95	3-100	0-100	50-82	3-99	3-100	44-100	24-65
Average, %	39	87	84	59	37	87	78	59	21	87	53	72	42	87	87	49

Annotation: CTEPH: chronic thromboembolic pulmonary hypertension, non-severe PH: PVR ≤ 5 WU/400 dyn∗sec∗cm^{-5}, NPV: negative predictive value, PAH: pulmonary arterial hypertension, PH: pulmonary hypertension, PPV: positive predictive value, QRS axis associated with right heart strain: QRS axis > 90°, SIQIII type or SISIISIII type, Sens.: Sensitivity, severe PH: PVR > 5 WU/400 dyn∗sec∗cm^{-5}, SLI: Sokolow-Lyon index, Spec.: Specificity

Table S5. Optimal cut-offs of electrocardiographic parameters for the diagnosis of pulmonary hypertension.

	Current cut-off	Optimal cut-off	Sensitivity (%)	Specificity (%)	AUC (95% CI)	Accuracy
P wave amplitude in II	≥ 0.25 mV	≥ 0.20 mV	66	81	0.79 (0.74-0.84)	0.71
R amplitude in I	≤ 0.2 mV	≤ 0.59 mV	81	52	0.71 (0.65-0.77)	0.69
R amplitude in aVR	> 0.4 mV	> 0.56 mV	41	37	0.39 (0.32-0.47)	0.37
R amplitude in V1	> 0.6 mV	> 0.11 mV	80	70	0.81 (0.75-0.86)	0.79
R amplitude in V6	< 0.3 mV	< 0.49 mV	29	83	0.53 (0.45-0.62)	0.45
S amplitude in V1	< 0.2 mV	< 0.44 mV	76	65	0.74 (0.67-0.82)	0.69
S amplitude in V5	> 1.0 mV	> 0.23 mV	80	60	0.75 (0.68-0.81)	0.71
S amplitude in V6	> 0.3 mV	> 0.21 mV	48	89	0.74 (0.67-0.80)	0.66
R/S in V1	> 1.0	> 0.25	75	73	0.83 (0.77-0.88)	0.75
R/S in V5	< 0.75	< 1.24	39	85	0.64 (0.55-0.72)	0.55
R/S in V6	< 0.4	< 4.19	76	67	0.73 (0.65-0.80)	0.76
R/S V5: R/S V1	< 0.04	< 4.30	76	82	0.84 (0.78-0.89)	0.74
(RI + SIII) − (SI + RIII)	< 1.5 mV	< -0.05 mV	76	92	0.86 (0.81-0.91)	0.78
R V1, V2 + S I, aVL - S V1	> 0.6 mV	> 0.12 mV	75	80	0.84 (0.79-0.89)	0.80
R V1, V2 + S I, V6 - S V1	> 0.6 mV	> 0.05 mV	75	73	0.82 (0.76-0.87)	0.77
R V1 + S V5, V6	> 1.05 mV	> 0.51 mV	69	84	0.82 (0.77-0.88)	0.77
Time to R peak in V1 (QRS < 120 ms)	> 35 ms	> 33 ms	66	86	0.75 (0.68-0.82)	0.75

Annotation: AUC: area under the curve, 95% CI: 95% confidence interval

Table S6. Patient characteristics and electrocardiographic data of patients with "R V1, V2 + SI, aVL − S V1" > 0.6 and ≤ 0.6 mV in pulmonary hypertension.

	> 0.6 mV	≤ 0.6 mV	*p*-value
n	103 (51%)	97 (49%)	-
sex, m/f, *n*	57, 46 (55%, 45%)	34, 63 (35%, 65%)	0.005
age, years, median (IQR)	62.4 (16.7)	68.9 (19)	0.015
PAH, *n*	51 (50%)	49 (51%)	1.000
IPAH, *n*	36 (71%)	37 (76%)	0.655
PAH-CTD, *n*	9 (18%)	6 (12%)	0.578
PoPH, *n*	5 (10%)	6 (12%)	0.758
PAH-HIV, *n*	1 (2%)	0 (0%)	1.000
CTEPH, *n*	52 (51%)	48 (50%)	1.000
Severe pulmonary hypertension	95 (93%)	65 (67%)	< 0.001
RAP, mm Hg, median (IQR)	9 (7)	7 (3)	0.022
mPAP, mm Hg, median (IQR)	47 (17)	40 (15)	< 0.001
CI, L/min/m^2, median (IQR)	2.3 (0.8)	2.5 (0.7)	0.002
CO, L/min, median (IQR)	4.1 (1.5)	4.2 (1.5)	0.022
PVR, dyn∗sec∗cm^{-5}, median (IQR)	684 (347)	510 (373)	< 0.001
NYHA I, *n*	0 (0%)	0 (0%)	-
NYHA II, *n*	8 (8%)	7 (7%)	1.000

NYHA III, *n*	82 (80%)	78 (80%)	1.000
NYHA IV, *n*	13 (13%)	12 (12%)	1.000
NT-pro-BNP, pg/ml, median (IQR)	1108 (1554)[+]	210 (951)[+]	< 0.001
BNP, pg/ml, median (IQR)	391 (698)[#]	189 (290)[#]	0.003
6 minute walk distance, m	360 (168)	378 (144)	0.168
Low risk (three-strata model)	1 (2%)[#]	6 (12%)[#]	0.057
Intermediate risk (three-strata model)	38 (75%)[#]	40 (82%)[#]	0.472
High risk (three-strata model)	12 (24%)[#]	3 (6%)[#]	0.023
BPA, *n*	52 (100%)[+]	48 (100%)[+]	1.000
Initial pharmacological monotherapy, *n*	29 (57%)[#]	36 (74%)[#]	0.099
Initial pharmacological dual therapy, *n*	20 (39%)[#]	11 (23%)[#]	0.157
Initial pharmacological triple therapy, *n*	1 (2%)[#]	0 (0%)[#]	0.805
QRS axis associated with right heart strain, *n* (%)	84 (82%)	36 (37%)	< 0.001*
P wave amplitude in II, mV, median (IQR)	0.20 (0.12)	0.20 (0.08)	0.002*
P wave amplitude in II ≥ 0.25 mV, *n* (%)	35 (34%)	15 (16%)	0.003*
P dextroatriale or biatriale, *n* (%)	56 (58%)	24 (26%)	< 0.001*
Right ventricular or biventricular hypertrophy (SLI), *n* (%)	71 (69%)	19 (20%)	< 0.001*
qR pattern in V1, *n* (%)	20 (19%)	7 (7%)	0.013
Right bundle branch block, *n* (%)	49 (48%)	20 (21%)	< 0.001*

R amplitude in V1, mV, median (IQR)	0.50 (0.37)	0.15 (0.15)	< 0.001*
R amplitude in V1 > 0.6 mV, *n* (%)	35 (34%)	0 (0%)	< 0.001*
S amplitude in V6, mV, median (IQR)	0.35 (0.36)	0.16 (0.25)	< 0.001*
S amplitude in V6 > 0.3 mV, *n* (%)	60 (58%)	22 (23%)	< 0.001*
R/S in V1, median (IQR)	2.33 (3.17)	0.33 (0.47)	< 0.001*
R/S in V1 > 1.0, *n* (%)	63 (70%)	11 (12%)	< 0.001*
R V1, V2 + S I, aVL - S V1, mV, median (IQR)	1.05 (0.67)	0.25 (0.54)	< 0.001*
R V1, V2 + S I, aVL - S V1 > 0.6 mV, *n* (%)	103 (100%)	0 (0%)	< 0.001*
R V1, V2 + S I, V6 - S V1, mV, median (IQR)	1.10 (0.66)	0.15 (0.55)	< 0.001*
R V1, V2 + S I, V6 - S V1 > 0.6 mV, *n* (%)	97 (94%)	8 (8%)	< 0.001*
R V1 + S V5, V6, mV, median (IQR)	1.08 (0.73)	0.58 (0.42)	< 0.001*
R V1 + S V5, V6 > 1.05 mV, *n* (%)	52 (51%)	12 (12%)	< 0.001*
Time to R peak in V1 (QRS < 120 ms), ms, median (IQR)	55 (20)	35 (44)	0.002*
Time to R peak in V1 (QRS < 120 ms) > 35 ms, *n* (%)	62 (77%)	42 (46%)	< 0.001*

Annotation: $^{+}$: only CTEPH patients, $^{\#}$: only PAH patients, *: a *p*-value < 0.004 was considered statistically significant after Bonferroni correction, AUC: area under the curve, BNP: brain natriuretic peptide, BPA: balloon pulmonary angioplasty, CI: cardiac index, CO: cardiac output, CTEPH: chronic thromboembolic pulmonary hypertension, IPAH: idiopathic pulmonary arterial hypertension, IQR: interquartile range, mPAP: mean pulmonary arterial pressure, NYHA: New York Heart Association, PAH: pulmonary arterial hypertension, PAH-CTD: PAH associated with connective tissue disease, PAH-HIV: pulmonary arterial hypertension associated with an HIV infection, PoPH: portopulmonary hypertension, PVR: pulmonary vascular resistance, RAP: right atrial pressure, QRS axis associated with right heart strain: QRS axis > 90°, SIQIII type or SISIISIII type, severe PH: PVR > 5 WU/400 dyn∗sec∗cm^{-5}, SLI: Sokolow-Lyon index

Table S7. Definitions of and annotations on complex electrocardiographic parameters.

Electrocardiographic parameter	Definition/annotation
QRS axis associated with right heart strain	QRS axis > 90°, SISIISIII type or SIQIII type
Left axis deviation	QRS axis < 30°
Normal QRS axis	QRS axis = 30-90°
P wave dispersion, ms	Longest P wave duration – shortest P wave duration
PR interval, ms	Only measured when no PQ interval could be identified in limb leads
Right ventricular hypertrophy (Sokolow-Lyon index)	Highest R amplitude in V1 or V2 + deepest S amplitude in V5 or V6 > 1.05 mV
Biventricular hypertrophy (Sokolow-Lyon index)	Highest R amplitude in V1 or V2 + deepest S amplitude in V5 or V6 > 1.05 mV AND deepest S amplitude in V1 or V2 + highest R amplitude in V5 or V6 > 3.5 mV
R V1, V2 + S I, aVL - S V1, mV	Highest R amplitude in V1 or V2 + deepest S amplitude in I or aVL – deepest S amplitude in V1
R V1, V2 + S I, V6 - S V1, mV	Highest R amplitude in V1 or V2 + deepest S amplitude in I or V6 – deepest S amplitude in V1
R V1 + S V5, V6, mV	Highest R amplitude in V1 + deepest S amplitude in V5 or V6
Time to R peak in V1 (QRS < 120 ms), ms	Time to the highest point of the R wave amplitude when QRS duration < 120 ms
T wave dispersion, ms	Longest T wave duration – shortest T wave duration
